# Tegaserod mimics the neurostimulatory glycan polysialic acid and promotes nervous system repair

**DOI:** 10.1016/j.neuropharm.2013.09.014

**Published:** 2013-09-22

**Authors:** J. Bushman, B. Mishra, M. Ezra, S. Gul, C. Schulze, S. Chaudhury, D. Ripoll, A. Wallqvist, J. Kohn, M. Schachner, G. Loers

**Affiliations:** a New Jersey Center for Biomaterials, Rutgers University, Piscataway, NJ 08854, USA; b Center for Molecular Neurobiology, University Medical Center Hamburg-Eppendorf, D-20246 Hamburg, Germany; c European ScreeningPort GmbH, Schnackenburgalle 114, D-22525 Hamburg, Germany; d DoD Biotechnology High Performance Computing Software Applications Institute, Telemedicine and Advanced Technology Research Center, US Army Medical Research and Materiel Command, Fort Detrick, MD 21702, USA; e Keck Center for Collaborative Neurosciences, Rutgers University, Piscataway, NJ 08854, USA

**Keywords:** Polysialic acid, Tegaserod, Mimetic, Peripheral nerve, Regeneration, Glycan, Drug repurposing

## Abstract

Glycans attached to the cell surface via proteins or lipids or exposed in the extracellular matrix affect many cellular processes, including neuritogenesis, cell survival and migration, as well as synaptic activity and plasticity. These functions make glycans attractive molecules for stimulating repair of the injured nervous system. Yet, glycans are often difficult to synthesize or isolate and have the disadvantage to be unstable in a complex tissue environment. To circumvent these issues, we have screened a library of small organic compounds to search for structural and functional mimetics of the neurostimulatory glycan polysialic acid (PSA) and identified the 5-HT_4_ receptor agonist tegaserod as a PSA mimetic. The PSA mimicking activity of tegaserod was shown in cultures of central and peripheral nervous system cells of the mouse and found to be independent of its described function as a serotonin (5-HT_4_) receptor agonist. In an *in vivo* model for peripheral nerve regeneration, mice receiving tegaserod at the site of injury showed enhanced recovery compared to control mice receiving vehicle control as evidenced by functional measurements and histology. These data indicate that tegaserod could be repurposed for treatment of nervous system injuries and underscores the potential of using small molecules as mimetics of neurostimulatory glycans.

## 1. Introduction

Polysialic acid (PSA) is a homopolymer of α-(2,8)-linked sialic acid residues numbering up to 200 in length, and is attached predominantly to the neural cell adhesion molecule (NCAM) (Finne et al., 1983; Muhlenhoff et al., 1996). PSA is expressed in the developing and adult nervous system of vertebrates, with expression localized to migrating cells, processes of neurons and glial cells, synapses and stem cells (Angata and Fukuda, 2010; Durbec and Cremer, 2001; Roche et al., 1997). PSA has been suggested to promote cell motility in the nervous system by primarily expanding the extracellular space due to its large hydration volume (Yang et al., 1994) and decreasing homophilic interactions of NCAM (Durbec and Cremer, 2001). PSA also mediates interactions of NCAM with heparan sulfate proteoglycans (Storms and Rutishauser, 1998), brain derived neurotrophic factor (Muller et al., 2000), α-amino-3-hydroxy-5-methyl-4-isoxazolepropionic acid receptors (Vaithianathan et al., 2004), N-methyl-d-aspartate receptors (Hammond et al., 2006; Senkov et al., 2006), histone H1 (Mishra et al., 2010) and myristoylated alanine-rich C kinase substrate (Theis et al., 2013).

The cell motility promoting effects of PSA are of interest for the treatment of nervous system injuries and disorders. Viral-induced expression of PSA enhances regeneration after spinal cord injury, promotes sensory neuron integration into the injured spinal cord, and increases Purkinje cell dendrite formation following injury (Zhang et al., 2007a, 2007b, 2007c). Overexpression of PSA on astrocytes improves axonal extension across spinal cord injuries (El Maarouf et al., 2006), and transplanted Schwann cells overexpressing PSA augment repair in both spinal cord (Luo et al., 2011; Papastefanaki et al., 2007) and peripheral nerve (Jungnickel et al., 2012). However, continuously expressed PSA slows the rate of myelination *in vivo* (El Maarouf et al., 2006; Franceschini et al., 2004), and high PSA expression correlates with invasiveness and malignancy of cancers (Petridis et al., 2009; Tanaka et al., 2001), suggesting that a dose- and time-constrained approach must be considered.

As PSA is rapidly degraded by sialidases in the complex tissue environment (Franz et al., 2005; Martini et al., 1994; Nagai et al., 1989), peptide mimetics of PSA were identified and shown to act as true functional counterparts of PSA (Torregrossa et al., 2004). Linear and cyclic PSA mimetic peptides have improved functional recovery following peripheral nerve and spinal cord injuries in mice (Marino et al., 2009; Mehanna et al., 2010, 2009). Small organic molecule mimetics of PSA offer further advantages for the development and regulatory approval for therapies. To build upon previous advances in identifying PSA mimetics and to increase the translational potential, we have screened a library of small compounds for mimetics of PSA and identified tegaserod. Tegaserod is a drug that received clinical approval for treatment of irritable bowel syndrome and constipation (Muller-Lissner et al., 2001) by stimulating 5-HT_4_ receptors on enteric neurons (Liu et al., 2005, 2009). The present study shows that tegaserod has a second and distinct mechanism of action as a small organic mimetic molecule of PSA.

## 2. Materials and methods

### 2.1. Competition ELISA with small organic compounds

To identify small molecule PSA mimetics the NIH Clinical Collection 1 Library was screened using methods similar to those previously used by our group (Loers et al., 2013; Torregrossa et al., 2004). Briefly, the PSA mimicking peptide, NTHTDPYIYPIDC (Mehanna et al., 2009), coupled to catalase was immobilized on the surface of 384-well plates (3 µg/ml; 25 µl/well; overnight at 4 °C). Ten micromolar of molecules from the library were incubated with 0.1 µg/ml and 25 µl/well of the PSA-specific monoclonal antibody 735 (Frosch et al., 1985) for 1 h at room temperature and then added to the wells. An HRP-coupled secondary antibody (1:5000 in PBS; Jackson ImmunoResearch) and ortho-phenylenediamine (0.5 mg/ml, 5 min; Thermo Scientific) as HRP substrate were used to assess antibody binding at room temperature. Binding was quantified using an ELISA reader (490 nm; µQuant, Bio-TEK) and the software KCjunior (Bio-TEK). The PSA peptide mimetic was used as a positive control, and experiments were repeated three times to identify true hits.

Following the initial screen, a competition ELISA was performed with varying doses of tegaserod maleate (Sequoia Research Products Ltd.) and the negative control compound nitrendipine (Sequoia Research Products Ltd.). The PSA peptide mimetic coupled to catalase was immobilized, and wells were incubated with increasing concentrations of tegaserod and nitrendipine, pre-incubated with antibody 735 (0.1 µg/ml) for 1 h at room temperature.

### 2.2. Surface plasmon resonance (SPR)

Binding affinities of organic compounds to the antibody 735 were evaluated in a competition experiment by SPR measurements carried out on a BIAcore 3000 instrument (GE Healthcare Europe GmbH) with sensor chips maintained at 25 °C for all experimental steps (Schulze, 2000). The running buffer was phosphate buffered saline, pH 7.3 (PBS). PSA mimicking peptide coupled to catalase was covalently immobilized to CM5 sensor chips (carboxymethyl dextran; GE Healthcare Europe GmbH) via primary amino groups, using standard coupling protocols (Karlsson et al., 1991). In brief, the sensor surface was activated by a 7 min pulse of 0.2 MN-ethyl-N-(3-dimethylaminopropyl) carbodiimide and 50 mM N-hydroxysuccinimide. The PSA mimetic peptide solution (10 nM in 10 mM sodium acetate, pH 5.2) was then injected for 5–10 min. Ethanolamine (1 M, pH 8.5) was used to block remaining activated carboxyl groups (1 h). Ligand densities of 100–150 fmol/mm^2^ were reached. Immobilized control peptide (10 nM PSA scrambled peptide mimetic coupled to catalase) was used as a reference surface. Regeneration of the sensor chip was achieved by injection of 10 mM glycine, pH 2.5, at 10 ml/min (two 30 s pulses). The data were analyzed using the BIA evaluation 3.0 software. All sensorgrams were corrected for background and bulk refractive index by subtraction of the reference.

To confirm that tegaserod binds specifically to antibody 735, a competition experiment was performed. First, the antibody (10 nM) was pre-incubated for 1 h at room temperature with different molar concentrations (1, 2, 5, 8, 15 and 30 µM) of tegaserod or the negative control compound nitrendipine. Then, the antibody/organic compound solutions were injected (1 ml/min) to the PSA peptide mimetic-coupled chip and binding was determined over 30 min. The surface of the sensor chip was subsequently regenerated with 10 mM glycine, pH 2.5, at 10 ml/min (three 30 s pulses).

### 2.3. Molecular modeling of tegaserod with the PSA-specific antibody 735

A model of PSA bound to the binding pocket of the antibody 735 was constructed using previous information (Evans et al., 1995). The 3-dimensional coordinates for antibody 735 were obtained from the Protein Data Bank (PDB id: 1PLG). A decamer of PSA was built using the program Discovery Studio (Accelrys Inc.) in helical conformation making approximately 1/2 of a turn within 17 Å (*n* = 6 residues per turn). Subsequently, we used the program PYMOL (Schrödinger Inc.) to produce a model of an eight-residue segment of PSA docked onto the antibody 735. The resulting model was manipulated in PYMOL to reproduce the specific intermolecular contacts between PSA and antibody 735 that had been identified (Evans et al., 1995). The PSA conformation and pose was then energy-minimized in complex with antibody 735 using Molecular Operating Environment (Chemical Computing Group) to construct the final model.

We carried out ligand docking of tegaserod with antibody 735. The crystal structure of antibody 735 was used for docking by isolating the immunoglobulin domains corresponding to the variable regions of the heavy and light chain of antibody 735, followed by hydrogen placement and energy minimization. We used Schrödinger’s Glide ligand-docking software (Friesner et al., 2004) to manually construct a 12 Å cubic docking grid that included the entire complementary determining region (CDR) of antibody 735. We prepared tegaserod using Schrödinger’s Ligprep software and carried out docking using Glide in standard precision mode. The top-ranked ligand pose was selected for further analysis.

### 2.4. In vitro analysis of neurite/process outgrowth

Primary cultures of cerebellar granule neurons (cGNs), dorsal root ganglion (DRG) neurons or Schwann cells were prepared from cerebella or dorsal root ganglia of 7-day-old (P7) C57BL/6J wild type mice as described (Kleene et al., 2001; Loers et al., 2005; Mehanna et al., 2009) and motoneurons were prepared from C57BL/6J wild type or NCAM^−/−^ 14-day-old (E14) mouse embryos as described (Simova et al., 2006). In brief, 48-well plates were coated with 0.01% poly-l-lysine (PLL) or poly-l-ornithine (PLO) overnight at 4 °C. Schwann cells, cerebellar neurons, DRG neurons (PLL) or motoneurons (PLO) were seeded at a density of 1.25 × 10^4^ (Schwann cells, DRG neurons and motoneurons) or 2.5 × 10^4^ cells (cGNs) per well in 250 µl of corresponding serum-free culture medium and compounds were added 1 h after seeding. After maintenance for 24 h at 37 °C, cells were fixed with 2.5% glutaraldehyde and stained with 1% methylene blue/toluidine blue in 1% borax. Morphological quantification of neurite or process lengths was performed as described (Mehanna et al., 2009). Schwann cell processes and neurites of cGNs, DRG neurons and motoneurons with a length of at least one cell body diameter were counted and total neurite or process length per cell was determined by counting 50 cells in each of two wells per experiment using an AxioVision system 4.6 (Carl Zeiss). At least three independent experiments were performed for each culture condition.

### 2.5. Effects of tegaserod on femoral nerve regeneration

#### 2.5.1. Mice

All experiments were conducted in accordance with the Rutgers Animal Care and Facilities Committee and the Institutional Animal Care and Use Committee (IACUC) and every effort was made to minimize animal suffering and the number of animals used in experiments. C57BL/6J wild type and NCAM^−/−^ mice were used for all experiments and kept under standard laboratory conditions with food and water supply *ad libitum* and with an artificial 12 h light/dark cycle. Twelve-week-old C57BL/6J mice were subjected to femoral nerve injury as described (Mehanna et al., 2009), but with tegaserod substituting for the PSA peptide mimetic. Briefly, Puramatrix hydrogel (BD Biosciences, Franklin Lakes, NJ) contained within a polyethylene conduit was used as the delivery vehicle for tegaserod. Hydrogels with and without tegaserod were induced to gel using 2× PBS as the gelation stimulant within the conduits, which were then sutured into the injured site. Vehicle consisted of the same hydrogel contained within the conduit, but without tegaserod.

#### 2.5.2. Determination of locomotor parameters

The foot base angle (FBA) was measured before surgery and every week following surgery before the sacrifice of animals as described (Mehanna et al., 2009; Simova et al., 2006). Single frame video motion analysis (Simi Reality Motion Systems, Simi Sportsplayer) was used to quantify the angle between the beam and the foot when the toes from the left leg are fully extended.

The protraction limb ratio (PLR) was similarly measured using single frame motion analysis (Irintchev et al., 2005) and is taken as an indicator of voluntary movement. Mice are suspended by the tail above a pencil and allowed to grasp the pencil. The PLR is measured by dividing the relative length that the uninjured hind leg extends to the pencil by the length that the injured hind limb extends.

To account for variabilities between animals, an index is calculated that takes into account the initial FBA and PLRs of animals before surgery. The recovery index is calculated in percent by the equation: RI = (*X*_pre_ − *X*_den_)/(*X*_den_ − *X*_reinn_) × 100, where *X*_pre_, *X*_den_ and *X*_reinn_ are values prior to operation, during the state of denervation (7 days after injury) and at the end-point of the study (here 42 days after injury), respectively. An RI value of 100 indicates complete recovery of locomotor function.

#### 2.5.3. Determination of quadriceps muscle mass

At the end of the locomotor measurements, the quadriceps muscles were removed from the injured and uninjured hind limbs. The muscle was bluntly dissected starting distally at the level of the patellar tendon, working proximally, and cut free from its attachments from the patellar tendon distally and from the pelvis and femur proximally. Muscles were blotted dry using a paper towel and the mass was weighed using a Mettler Toledo XS105 Dual Range analytical balance.

#### 2.5.4. Histology

Animals were perfused with 4% paraformaldehyde and explanted nerves were post-fixed in osmium tetroxide and embedded in resin as described (Masand et al., 2012). One and 2 µm thick cross sections were cut midway into the region of the regenerated nerve within the conduits, and sections were stained with 1% toluidine blue and 1% borax. Sections were imaged with a Zeiss Axiocam, using 20×, 40×, and 100× objectives. ImageJ software was used to calculate the number of axons as described (Mehanna et al., 2009).

## 3. Results

### 3.1. Identification of tegaserod as potential mimetic of PSA

**Fig. 1:**
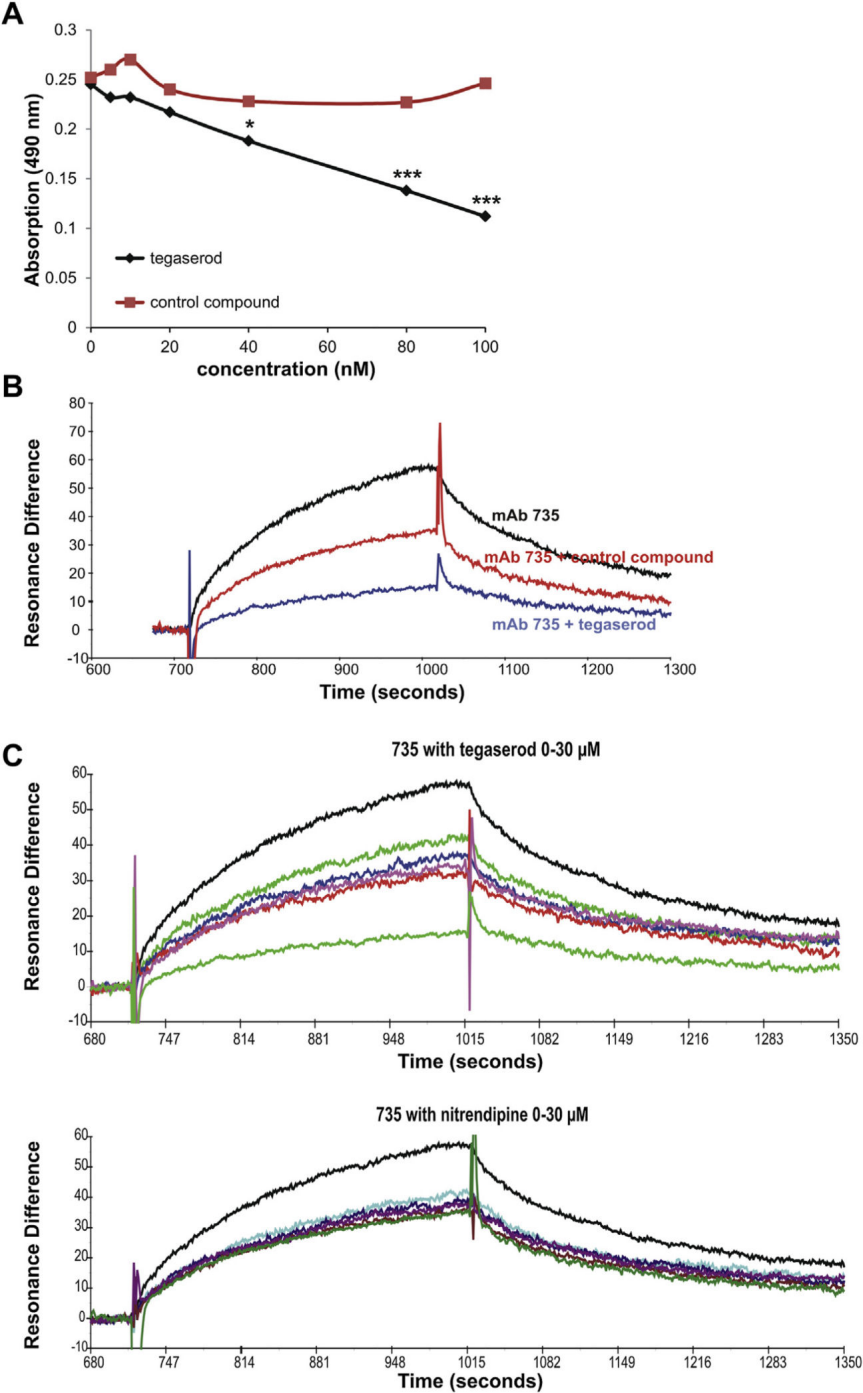
Tegaserod competes with PSA peptide mimetic for binding to the PSA-specific antibody 735 (mAb 735). (**A**) Results from a competition ELISA of the dose-dependent ability of tegaserod to interfere with binding of the antibody 735 with the PSA peptide mimetic immobilized at the bottom of the wells, *p < 0.05; ***p < 0.0005 (data were compared by one-way analysis of variance (ANOVA)). (**B**) Surface plasmon resonance (SPR) profile of the ability of 100 nM of tegaserod to interfere with binding of antibody 735 with the PSA peptide mimetic covalently immobilized to a CM5 sensor chip. The SPR signal is displayed as resonance versus time where 1000 RU (resonance units) represent a shift in resonance angle of 0.1° corresponding to a change in surface antibody concentration of ~1 ng/mm^2^. (**C**) Surface plasmon resonance (SPR) profile of the ability of 0.1, 1, 10, 20 and 30 µM tegaserod or nitrendipine to interfere with binding of antibody 735 with the PSA peptide mimetic. Only tegaserod interferes with binding of antibody 735 with the PSA peptide mimetic in a concentration dependent manner.

NIH Clinical Collection 1 Library was screened via competition ELISA for compounds that inhibit binding of the PSA receptor site of antibody 735 to a previously identified peptide mimetic of PSA (Mehanna et al., 2009). This screen resulted in the identification of six drugs, one of which was tegaserod. To confirm the results of the initial screen, a second competition ELISA was performed with a range of tegaserod concentrations. Tegaserod inhibited binding of antibody 735 to the PSA peptide mimetic in a dose dependent manner (Fig. 1A), with maximal inhibition at approximately 100 nM. The ability of tegaserod to inhibit binding of the PSA peptide mimetic to antibody 735 was compared against the control compound nitrendipine, which did not impede antibody binding at any concentration.

The capacity of tegaserod to interact with the antibody 735 antigen binding site was also evaluated by surface Plasmon resonance (SPR), which provides a more sensitive and kinetic platform to probe molecular interactions. Tegaserod disrupted binding of antibody 735 to the PSA peptide mimetic that was bound to the chip surface by 80% compared to antibody in the absence of tegaserod (Fig. 1B). Nitrendipine showed markedly less inhibition than tegaserod. In addition, a concentration dependent inhibition of binding between antibody 735 and PSA peptide mimetic could be shown by SPR in the presence of tegaserod, but not in the presence of the control compound nitrendipine (Fig. 1C).

### 3.2. Molecular modeling of tegaserod in the PSA receptor pocket

**Fig. 2:**
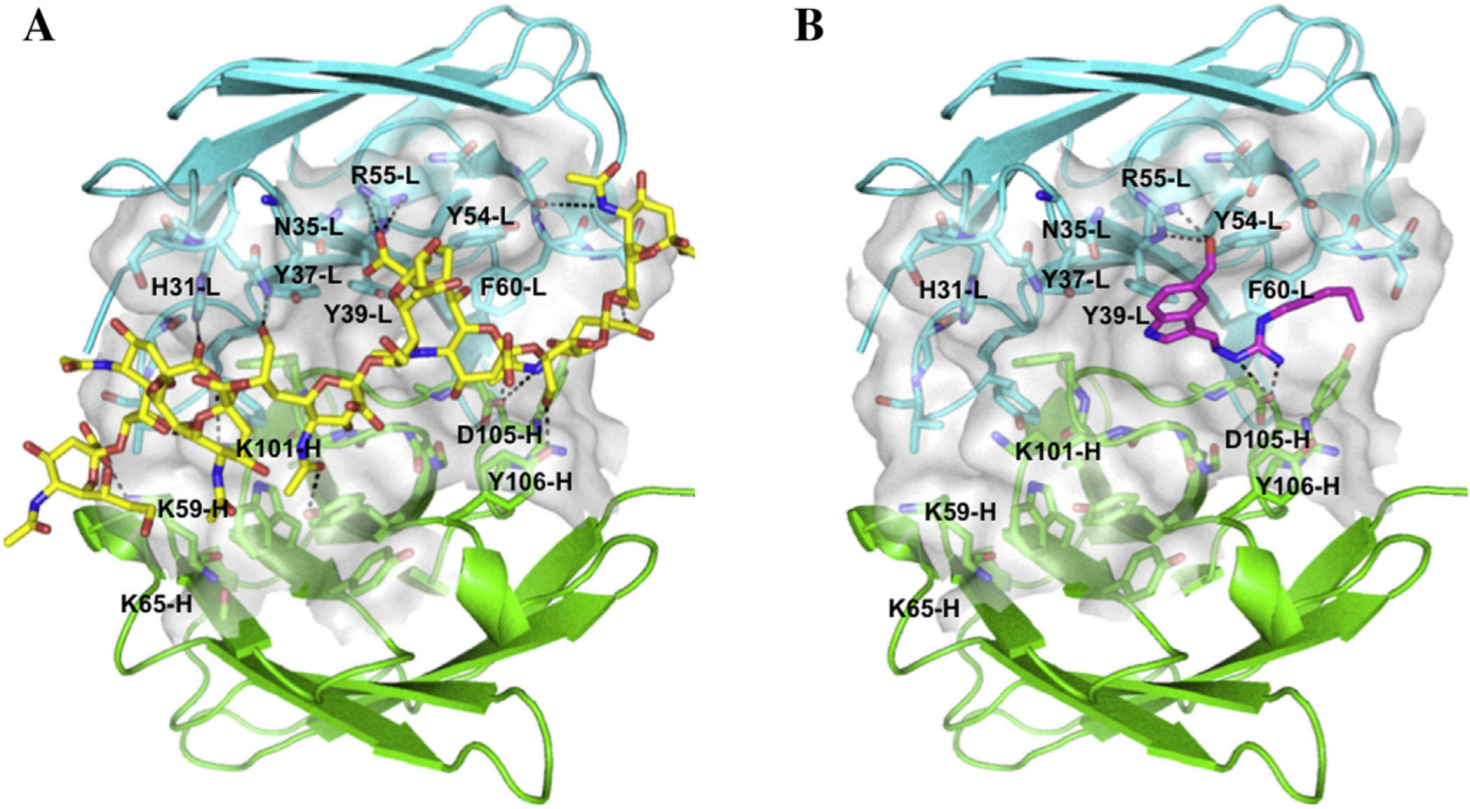
Structure models of PSA and tegaserod in complex with the surrogate PSA binding pocket of antibody 735 (heavy chain, green; light chain, cyan) derived from molecular modeling and docking. (**A**) PSA, depicted in yellow, binds to a broad groove in the antibody 735 CDR region forming multiple hydrogen bonds. (**B**) Tegaserod, depicted in magenta, binds within a groove in one region of the putative PSA-binding site, where it is anchored primarily by a salt-bridge formed between the guanidinium group of the ligand with aspartic acid 105 on the heavy chain located deep within the groove. (For interpretation of the references to color in this figure legend, the reader is referred to the web version of this article.)

To identify how a small molecule such as tegaserod could act as a mimetic for the larger and negatively charged PSA, molecular modeling of PSA and tegaserod was performed within the published structure for the PSA binding pocket of antibody 735 (Evans et al.,1995). The broad complementary determining region (CDR) of antibody 735 contains two biochemically distinct regions: a hydrophobic region rich in aromatic residues and several polar residues, and a positively charged region consisting of multiple lysine residues. An eight-residue PSA fragment forms a half-helical turn that binds within a broad cleft in the antibody CDR region (Fig. 2A). PSA residues 1–4, which expose N-acetyl groups to the antibody surface bind to the hydrophobic region of the CDR and form hydrogen bonds with aspartic acid (D)105 – heavy chain (H) and arginine (R)55 – light chain (L). These interactions are thought to be critical to the specificity of antibody 735 for N-acetyl over N-propyl PSA. By contrast, PSA residues 5–8 present largely negatively charged carboxyl groups to the positively charged region of the CDR, forming salt-bridges with lysine (K)65-H and K101-H. Overall, the model confirms previous observations (Evans et al., 1995), in that the aromatic/polar region of the CDR is responsible for N-acetyl PSA specificity, while the positively charged region forms more promiscuous interactions with multiple negatively charged groups in PSA.

Using the ligand docking software Glide (Friesner et al., 2004), tegaserod was docked into the CDR region of antibody 735 without further modifications to the receptor structure. The top-ranked pose (Fig. 2B) shows tegaserod binding to the hydrophobic region of the antibody CDR, forming hydrogen bonds with two key residues, D105-L and R55-H, that also mediate PSA binding. The pose suggests that tegaserod may be further stabilized by cation-∐ interactions between R55-H and the tegaserod ring as well as aromatic interactions with numerous residues in the binding site such as tyrosine (Y)37-L, Y54-L, Y32-H, Y33-H, and phenylalanine (F)102-H. These docking results suggest that tegaserod competes with PSA by binding to a hydrophobic groove within the PSA-binding site of antibody 735 and reproducing a number of intermolecular hydrogen bonds found in the antibody 735 complex model.

### 3.3. In vitro activity of tegaserod

**Fig. 3:**
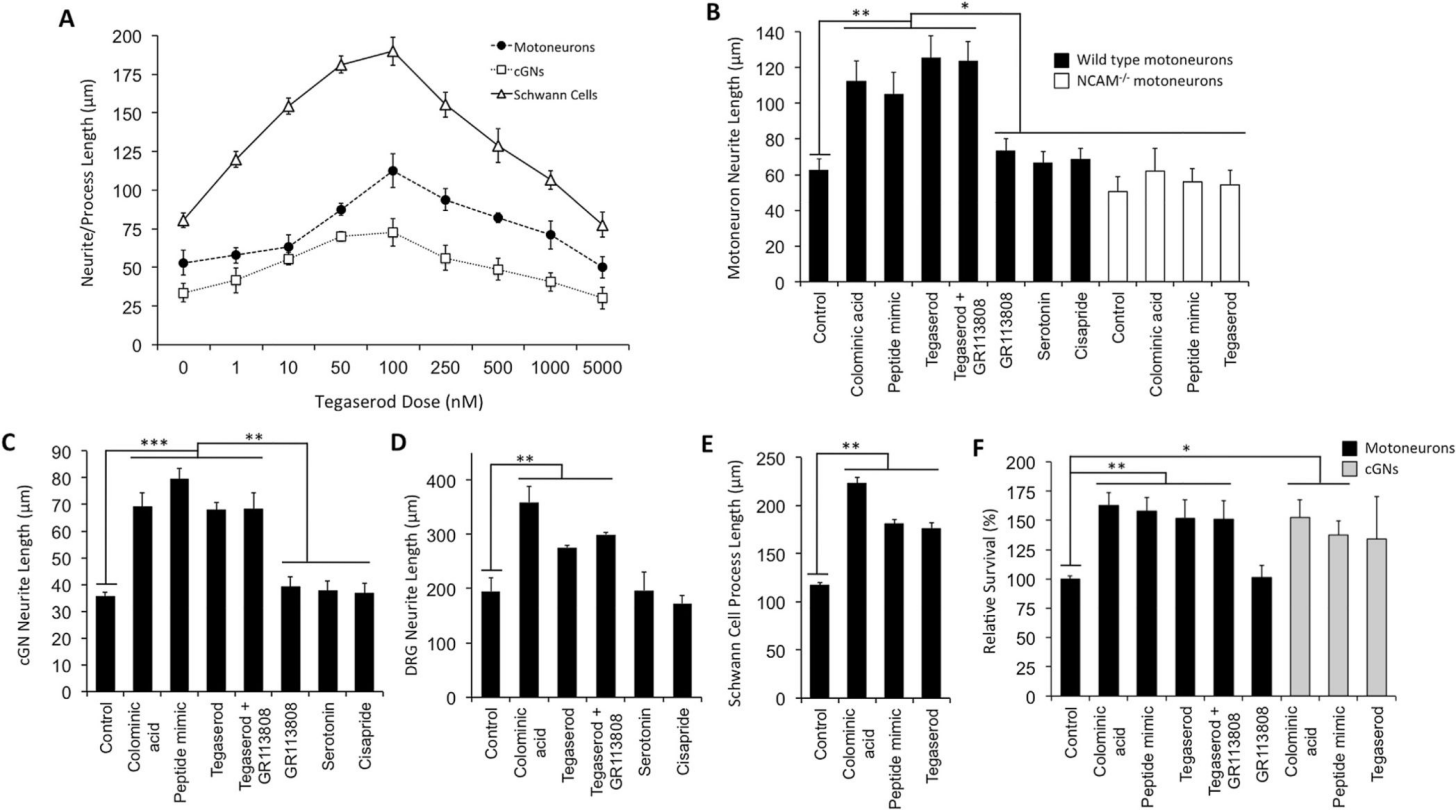
*In vitro* activity of tegaserod on extension of neurites/processes from and survival of motoneurons, cerebellar granule neurons (cGNs), dorsal root ganglion (DRG) neurons and Schwann cells. (**A**) Neurite/process extension following varying doses of tegaserod after 24 h. (**B**) Comparison of the neurite-extending capabilities of tegaserod and related compounds on murine motoneurons from wild type and NCAM deficient (NCAM^−/−^) mice. Tegaserod elicits neurite extension comparable to the PSA analog colominic acid and the PSA peptide mimetic, which is not observed in motoneurons from NCAM^−/−^ mice. 5-HT_4_ agonists serotonin and cisapride as well as the 5-HT_4_ antagonist GR113808 do not increase neurite extension from motoneurons, nor did GR113808 attenuate the effects of tegaserod. (**C–E**) Effects of tegaserod and related compounds on cGNs, DRG neurons and Schwann cells. (**F**) Relative survival of motoneurons and cGNs treated with tegaserod and related molecules. Concentrations of compounds, glycans and peptides: tegaserod, serotonin, cisapride and GR113808 (100 nM), colominic acid (3 µM), PSA peptide mimetic (30 µM). All treatments were performed in duplicates and at least 100 cells were counted for each treatment. Results are from two or more experiments. Mean values ± SEM are shown. (C–E) *p < 0.05, **p < 0.005, ***p < 0.0005 via Student’s *t*-test.

Functional activity of tegaserod was assessed *in vitro* using PSA-responsive murine cGNs, DRG neurons, Schwann cells, and motoneurons. Schwann cells, DRG neurons and motoneurons are key cell types involved in peripheral nerve regeneration and muscle reinnervation and cGNs were chosen because they are, like motoneurons and Schwann cells, responsive to PSA, but are known not to be responsive to serotonin (5-hydroxytryptamine; 5-HT) for neurite extension (Zilkha-Falb et al., 1997). A dose–response curve of tegaserod for motoneurons, Schwann cells and cGNs, and revealed that tegaserod affects neurite and process extension of neurons and Schwann cells, respectively, at 1 nM and reached an optimal effect at 100 nM for all cell types (Fig. 3A). At doses exceeding 100 nM, the effects of tegaserod on increasing neurite and process extension began to decline and reached control levels between 1 µM and 5 µM.

Experiments were next conducted to compare the activity of tegaserod with the PSA peptide mimetic and the bacterial PSA analog colominic acid on motoneuron neurite extension. Colominic acid, the PSA peptide mimetic, and tegaserod all stimulated neurite extension of murine motoneurons, while cisapride (another 5-HT_4_ agonist (Quigley, 2011)) and serotonin did not enhance motoneuron neurite extension (Fig. 3B). Furthermore, the 5-HT_4_ receptor antagonist GR113808 (Gale et al., 1994) did not inhibit motoneuron extension and co-treatment of neurons with tegaserod and GR113808 did not decrease the stimulatory effect of tegaserod. Importantly, motoneurons isolated from NCAM^−/−^ mice were not enhanced in neurite extension by colominic acid, the PSA peptide mimetic, or tegaserod, suggesting that tegaserod affects motoneurons via NCAM. Similarly, the effects of tegaserod were comparable to those of colominic acid and the PSA mimetic peptide on cGNs, DRG neurons and Schwann cell neurite/process extension (Fig. 3C, D, E). Neurite/process extension of these cell types could not be duplicated with serotonin or cisapride, and was not lowered by co-administration of GR113808 with tegaserod. Finally, exposure to tegaserod also increased survival of motoneurons and cGNs (Fig. 3F).

### 3.4. Effects of tegaserod on regeneration following femoral nerve injury

#### 3.4.1. Metrics of functional recovery

**Fig. 4:**
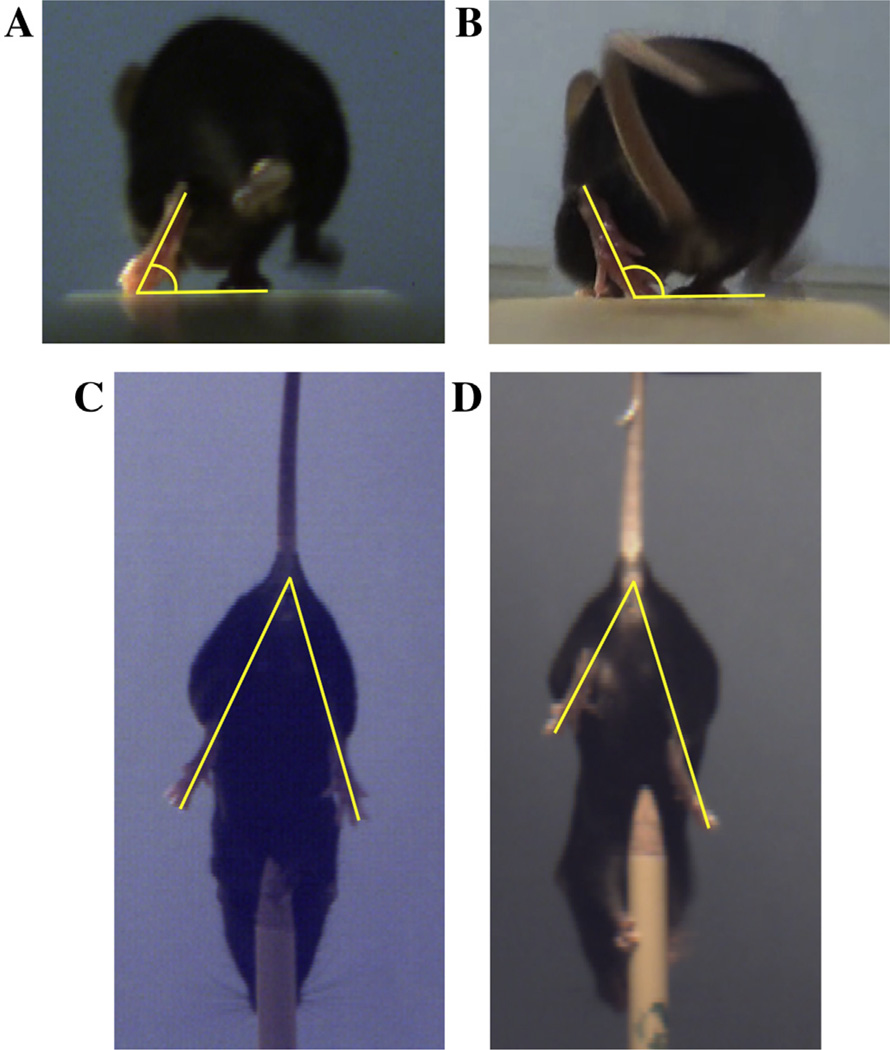
Metrics of functional recovery of limb function following femoral nerve injury. (**A–B**) The foot base angle (FBA) is the angle between the walking surface and the hind foot when the contralateral foot is lifted. (**A**) In an uninjured mouse, the FBA is 60–70°. (**B**) Following injury, the FBA of the injured leg increases to 100–110°. As the femoral nerve reinnervates the quadriceps muscle and muscle function is restored, the FBA decreases towards pre-injury levels. (**C–D**) The protraction limb ratio (PLR) measures the voluntary movement of the hind limbs to grasp an object when hanging upside down. The PLR measures the distance between the base of the tail to the tip of the extended limbs, as measured by dividing the length of the uninjured limb by that of the injured limb. (**C**) In uninjured animals, both hind limbs extend equal distances resulting in a PLR close to 1. (**D**) Following femoral nerve injury the limb with the injured nerve fails to extend to the same extent as the uninjured hind limb, giving a PLR of >1. A decline in the PLR toward 1 over time indicates recovery.

The *in vivo* efficacy of tegaserod was tested in the mouse femoral nerve injury model, with 250 nM or 2500 nM of tegaserod being encapsulated in the BD Puramatrix hydrogel within an inert conduit that was sutured between two nerve stumps with a gap of 2 mm length. The vehicle control group received the same hydrogel contained within the conduit but without tegaserod. The 2 mm gap length is sub-critical and limited recovery is expected in the control group. Locomotor recovery was quantified as described (Mehanna et al., 2009) through assessment of the foot base angle (FBA) and protraction limb ratio (PLR) as shown in Fig. 4. Studies utilizing this method have found maximal recovery of the FBAs between 75 and 85° (Irintchev et al., 2005; Masand et al., 2012; Mehanna et al., 2009).

#### 3.4.2. Functional recovery

**Fig. 5:**
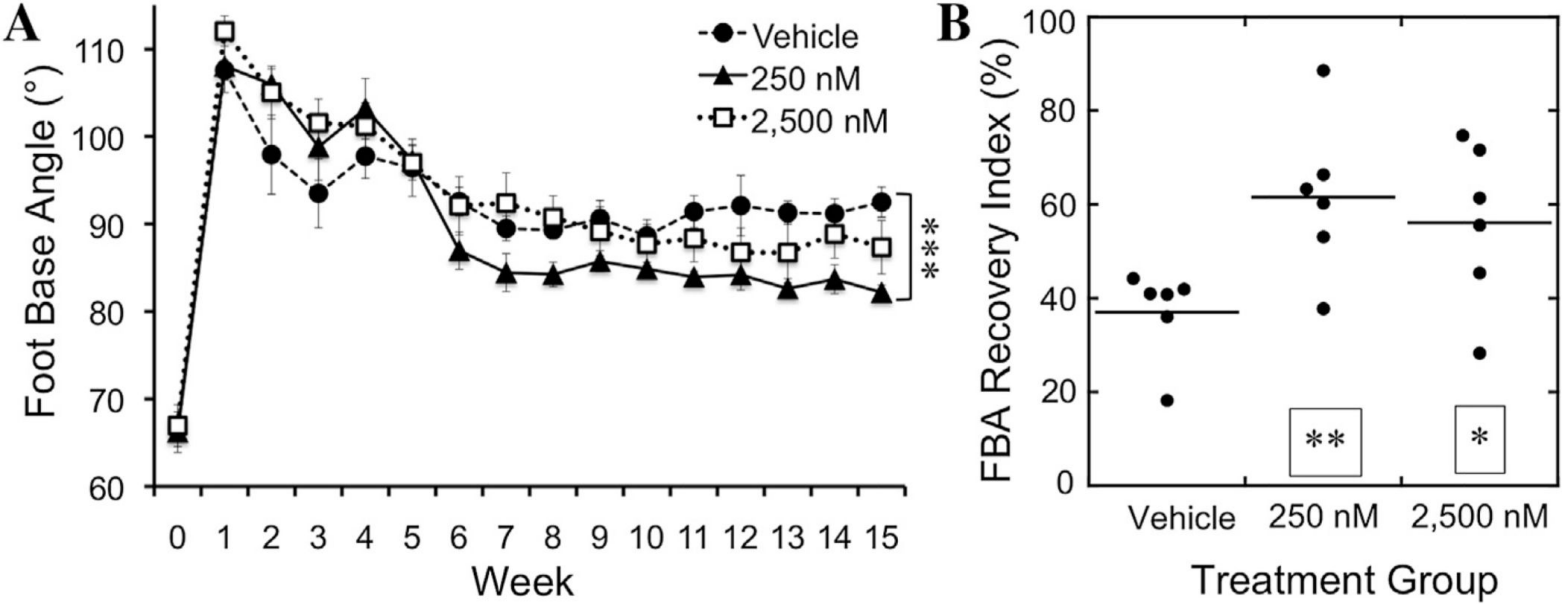
Evaluation of functional recovery of mice via measurement of the foot base angle (FBA) following femoral nerve injury and insertion of a conduit containing vehicle alone, 250 nM tegaserod, or 2,500 nM tegaserod. Pre-injury FBAs are between 60 and 70°, which increase following injury, and a return toward pre-injury angles indicates recovery. (**A**) FBA of mice that received vehicle, 250 nM tegaserod, or 2,500 nM tegaserod over a 15-week recovery period. ***p < 0.0005 via one-way ANOVA with Tukey’s post-hoc test. (**B**) Recovery index (RI) of the FBA of individual animals within each treatment group is shown by normalizing the recovery of individual animals to their pre-injury FBA, **p < 0.005, *p < 0.05 via one-way ANOVA with Tukey’s post-hoc test.

Analysis of the FBA showed that tegaserod improved this functional metric of regeneration. A sustained improvement of the FBA in mice receiving 250 nM tegaserod-containing conduits first became apparent 6 weeks after the injury and became statistically significantly different from the vehicle control group at 10 weeks (Fig. 5A). The level of recovery of the FBA from 250 nM tegaserod exceeds or is equivalent to the maximal recovery found in other studies using this model (Irintchev, 2011; Masand et al., 2012; Mehanna et al., 2009; Simova et al., 2006). Animals that received the higher dose of tegaserod, 2500 nM, tended toward improved FBA recovery but did not achieve a statistically significant difference from the vehicle control.

Given the variation between individual animals, a recovery index (RI) for the FBA was calculated which normalizes the recovery at the end of the 15-week assessments to the initial FBA and the FBA measured at week 1, which is within 5–7 days of the surgery. The RI is expressed as a percentage, with the vehicle control group showing an average RI of 37%, with 5 of the 6 animals clustered between 36% and 42% (Fig. 5B). Animals that had received 250 nM tegaserod recovered to an average of 62%, while animals that had received 2500 nM tegaserod showed an RI of 56%, with considerable variation for animals having received 2500 nM tegaserod.

**Fig. 6:**
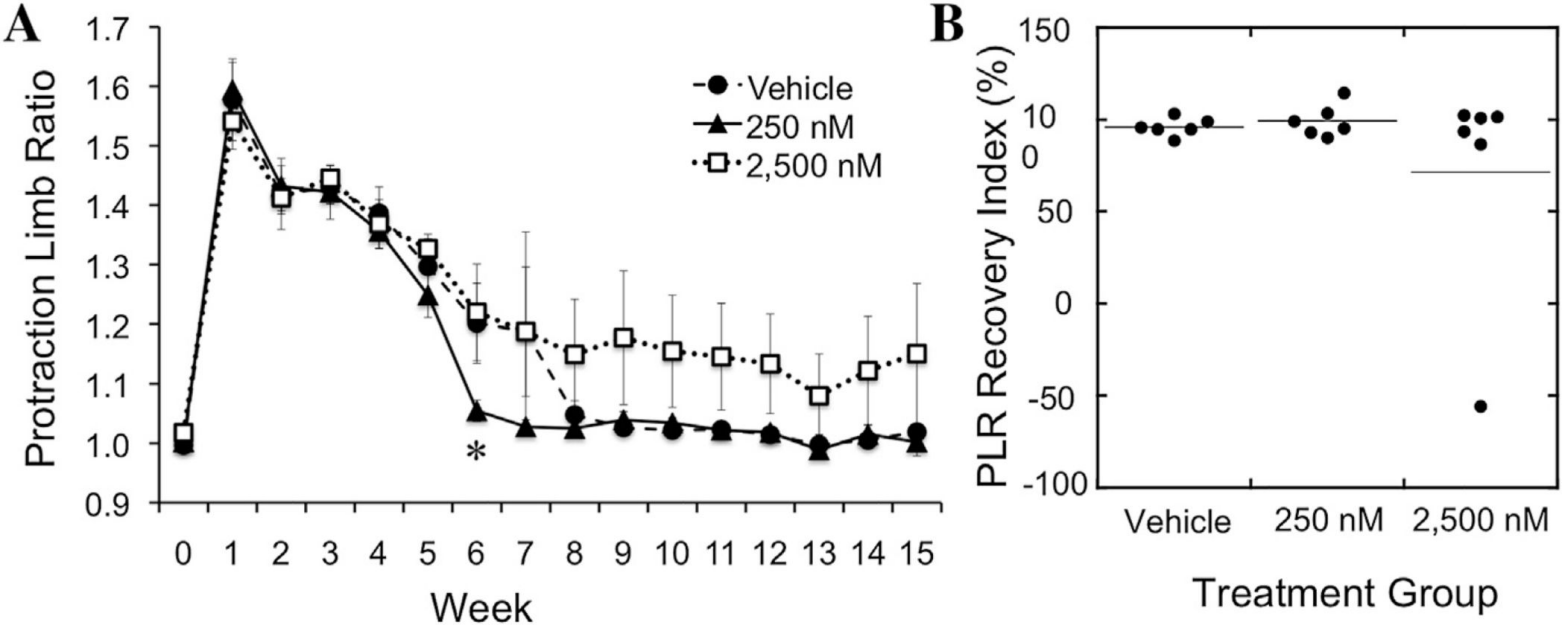
Evaluation of functional recovery via the protraction limb ratio (PLR) following femoral nerve injury. Pre-injury PLR of 1 is increased following injury as the injured limb cannot extend to the same extent as the uninjured limb. A return to 1 indicates recovery of function. (**A**) PLR of mice that received vehicle, 250 nM tegaserod, or 2,500 nM tegaserod measured over a 15-week recovery period. *p < 0.05 via one-way ANOVA with Tukey’s post-hoc test. (**B**) Recovery index (RI) of PLR of individual animals within each treatment group after 15 weeks.

In addition to the FBA measurements, the PLR was evaluated. Following injury, the injured leg does not extend to the same extent as the uninjured leg, but will do so as nerve function is restored (Fig. 4). Analysis of the PRL with and without tegaserod suggested a positive influence at a 250 nM tegaserod concentration, but not at 2500 nM (Fig. 6A). Vehicle control mice without tegaserod returned to pre-surgery PLR by 8 weeks, compared with 6 weeks for mice having received 250 nM tegaserod. In contrast, mice that had received 2500 nM tegaserod exhibited PLRs that remained higher than the other two groups. Calculation of the RI for each mouse revealed that, although the majority of mice with 2500 nM eventually did recover to an RI of 100%, one mouse within this group did not recover to pre-surgical PLR functional performance (Fig. 6B).

#### 3.4.3. Effect of tegaserod treatment on muscle mass

**Fig. 7:**
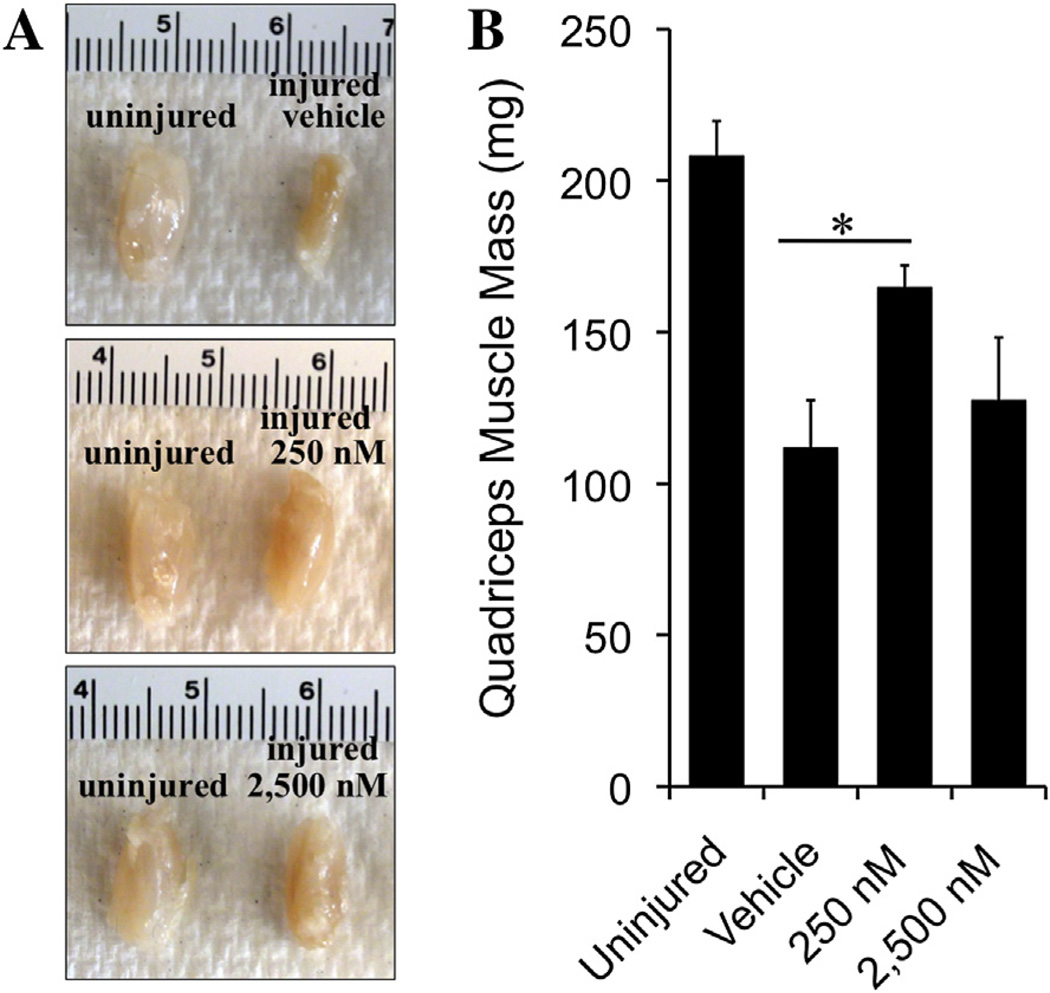
Mass of quadriceps muscle of mice after 15-week recovery period. (**A**) Images of representative quadriceps muscles from the healthy and injured hind limbs of mice following femoral nerve injury and insertion of a conduit containing vehicle or two doses of tegaserod. (**B**) Quantification of average quadriceps muscle masses of mice that received vehicle, 250 nM tegaserod, or 2,500 nM tegaserod, and the average muscle mass from the uninjured hind limbs. *p < 0.05 via Student’s *t*-test.

Muscles rapidly atrophy if nerve conduction is lost and will regain muscle mass as reinnervation occurs. The quadriceps muscle, which is solely innervated by the femoral nerve, was weighed at the end of the 15-week recovery period as an additional metric of recovery. The quadriceps muscle from the injured limbs of mice that had received 250 nM tegaserod showed a 50% increase in muscle mass compared to the quadriceps muscle of the control group (Fig. 7). Quadriceps muscles from mice having received 2500 nM tegaserod were slightly heavier when compared to the control group.

#### 3.4.4. Histological assessment of nerves

**Fig. 8:**
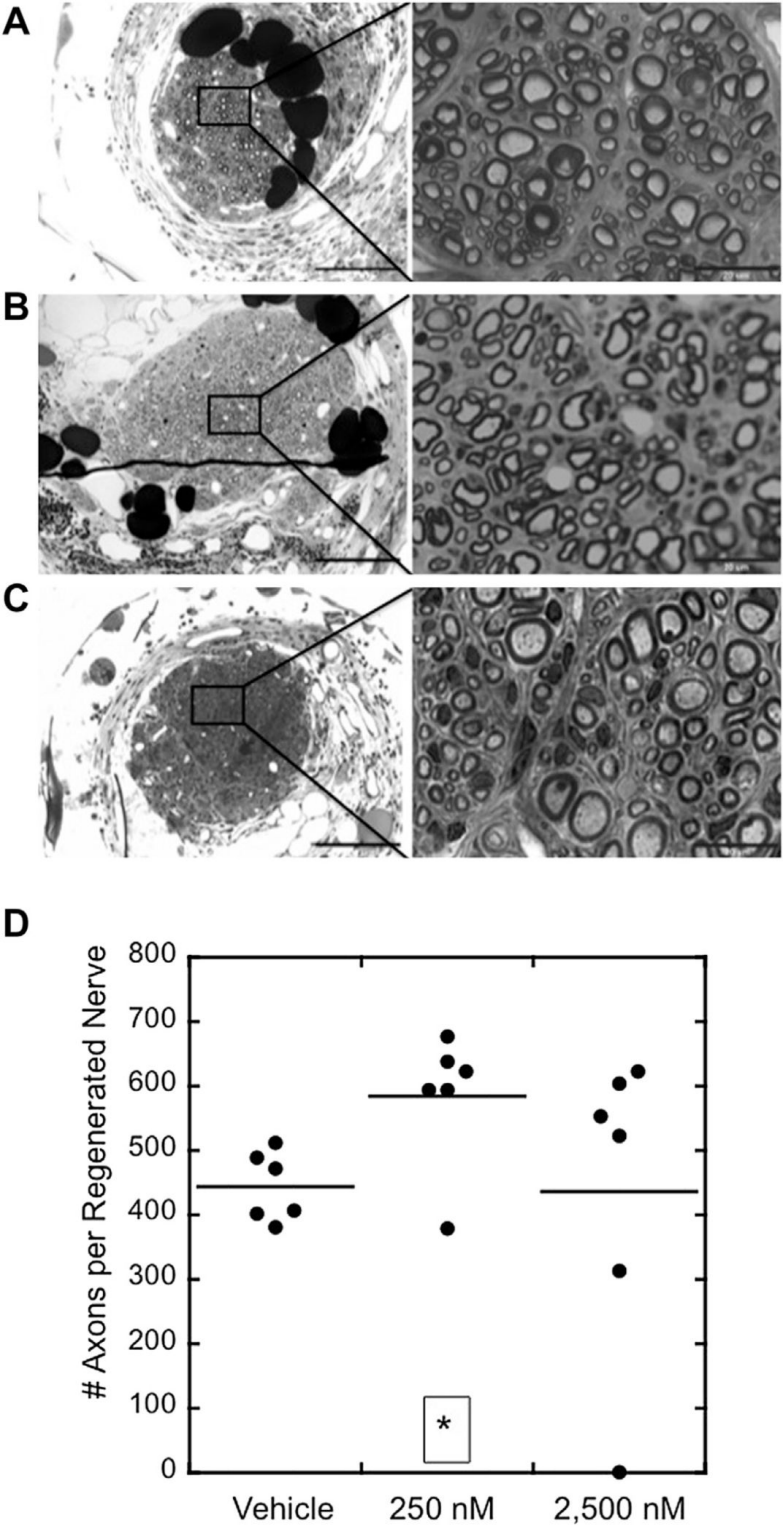
Histology of femoral nerves and quantification of regenerated axons after a 15-week recovery period. (**A–C**) 10× and 100× magnification images of regenerated nerves from animals treated with (**A**) vehicle control, (**B**) 250 nM tegaserod, and (**C**) 2,500 nM tegaserod. (**D**) Dot plot shows the numbers of axons in the center of the regenerated nerves and averages within groups. *p < 0.05 via Student’s *t*-test.

At the end of the recovery period, femoral nerves were fixed and histology was performed to assess the histology of the nerve 1 mm into the 2 mm gap (Fig. 8A–C). Regenerated nerves treated with vehicle control contained an average of 432 axons, compared to 589 and 434 for nerves treated with 250 nM and 2500 nM tegaserod, respectively (Fig. 8D). When the degree of myelination (in percent) and g-ratios of the myelinated axons were quantified from images obtained from light miscroscopy, no significant differences between groups were detectable (data not shown).

## 4. Discussion

Drug repurposing has the potential to rapidly introduce new therapies using existing drugs for novel applications. Previously approved drugs have known toxicological and pharmacokinetic profiles and thus have the potential to avoid a repetition of these time consuming and costly studies prior to gaining approval for a new application, provided that the therapeutic dose is equal to or less than the original application. In the majority of cases, a drug is repurposed for a different indication based on its known mechanism of action. For example, celecoxib was repurposed from treating osteoarthritis to familial adenomatous polyposis based on the activity of inhibiting cyclooxyenase-2 (Phillips et al., 2002). Other compounds, such as β-lactam antibiotics and minocycline, have been found to act via thus far undefined molecular mechanisms to upregulate expression of proteins that confer neuroprotection (Plane et al., 2010; Rothstein et al., 2005). We have, in contrast to these approaches, used an *in vitro* approach to investigate new functional mechanisms for a drug targeted to a different disease paradigm. We have thus been able to present what appears to be one of the first reports on a small molecule mimetic of a nervous system glycan.

The small organic compound tegaserod was identified as a PSA mimetic by a competition ELISA screen and *in vitro* assays confirmed its PSA mimicking effect on central and peripheral nervous system cells. Lack of activity on NCAM^−/−^ motoneurons and the inability of serotonin or other 5-HT_4_ agonists to replicate the activity of tegaserod *in vitro* indicate a structural mimicking activity consistent with the screening method for PSA mimetics and a functional activity independent of serotonin and/or serotonin receptors. While the molecular modeling studies and cell culture assays indicate that tegaserod may act via NCAM and its co-receptors, it is also conceivable that tegaserod binds to and acts via additional PSA receptors, such as heparin sulfate proteoglycans (Storms and Rutishauser, 1998), brain derived neurotrophic factor (Muller et al., 2000), excitatory amino acid receptors (Hammond et al., 2006; Kochlamazashvili et al., 2010, 2012; Vaithianathan et al., 2004), histone H1 (Mishra et al., 2010) and/or myristoylated alanine-rich kinase C substrate (Theis et al., 2013). Although the ability of tegaserod to interact with these molecules is yet unknown, it can plausibly be expected that, given the structural and functional similarity of tegaserod with oligomers within PSA, tegaserod will subserve many interactions of PSA.

It is noteworthy that tegaserod leads to maximal neurite extension *in vitro* at 1/10th and 1/100th of the doses required for the peptide mimetic and colominic acid, respectively. Other *in vitro* experiments with modified PSA derivatives used µM to mM PSA concentrations and PSA of different chain lengths (Berski et al., 2008; Haile et al., 2008). Although these results are not strictly comparable to the ones of the present study, they indicate that concentrations of native PSA in the in the higher µM range are needed for optimal effects.

In a previous study, the extent of regeneration induced by µM concentrations of the PSA peptide mimetic in the same injury model (Mehanna et al., 2009) was similar to that promoted by 250 nM tegaserod in the present study which showed that 250 nM of tegaserod beneficially acted in regeneration as indicated by several parameters measured *in vivo*. The level of recovery was superior or equivalent to that promoted by PSA and the PSA peptide mimetic in this and other peripheral nervous system regeneration paradigms (Gravvanis et al., 2007; Haastert-Talini et al., 2010; Jungnickel et al., 2009, 2012; Marino et al., 2009; Masand et al., 2012; Mehanna et al., 2009). While the proof-of-concept experiments of the present study are encouraging, it will be important to assess the ability of tegaserod to promote nerve repair in larger peripheral nerve gaps or other paradigms of regeneration in acutely and chronically injured mammalian nervous systems.

The effects of PSA, the peptide mimetic and colominic acid on neurite extension plateau at concentrations in the µM range while higher doses of tegaserod *in vitro* show a reduced level of stimulation and the highest doses were comparable to unstimulated control values. It is not uncommon for drugs to have an optimal dose range and exert undesirable effects if this dose range is exceeded. It is interesting in this context that overall cell viability was not reduced at higher doses; the reason for the function reducing effects on neurite extension at higher concentrations is presently unknown. It should be noted that the reduced average values of locomotor recovery observed *in vivo* at 2500 nM concentrations could be due to a single animal in this group where no axons were detected by histological analysis, thus raising the possibility that this dose mirrors the less beneficial effects seen at higher doses *in vitro*.

Like most agonists, tegaserod is not entirely specific for its cognate receptor, the 5-HT_4_ receptor and stimulates, although less efficiently, other serotonin receptors (Sanger, 2009; Smith et al., 2008). Of these receptors, 5-HT_2B_ has been reported in rat peripheral nerves and tegaserod’s pharmacological effect on human 5-HT_2B_ is only 10% of that achieved via 5-HT_4_ receptor (Beattie et al., 2004; Smith et al., 2008). The 5-HT_2B_ receptor in rat peripheral nerves is expressed by Schwann cells (Gaietta et al., 2003; Yoder et al., 1997) and triggering this receptor leads to Ca^2+^ release from Schwann cells *in vitro* (Yoder et al., 1996). How serotonin affects Schwann cell process extension or peripheral nerve regeneration has not been investigated.

Expression of 5-HT_4_ receptor mRNA in rat DRG neurons has been reported (Nicholson et al., 2003), but the functional implications of 5-HT_4_ actions in the PNS outside of the enteric nervous system remain to be determined. Our data indicate that the 5-HT_4_ receptor agonists serotonin and cisapride fail to promote neurite extension from DRG neurons, while tegaserod is effective and its action is not reduced by the 5-HT_4_ receptor antagonist GR113808. Despite these effects, it is unlikely that the improvements in functional motor control and muscle mass noted in our studies would be due to an effect of tegaserod on sensory axons. Sensory axons lack the intrinsic capability of stimulating muscles and those sensory axons that mistakenly reinervate into the motor branch following nerve injury are removed in a process called pruning (Brushart, 1993; Redett et al., 2005). However, while tegaserod has been found to stimulate neurite extension *in vitro* as PSA does via NCAM, we cannot rule out the possibility that tegaserod may be acting through other PSA receptors or additional indirect pathways *in vivo* to promote peripheral nerve regeneration.

Current FDA-approved conduits for the repair of peripheral nerves are reported to only be effective for gaps of ≤3 cm, and regeneration is often suboptimal for these smaller gap sizes (Kehoe et al., 2012). These in-market conduits provide mechanical and directional support, but are not biologically inductive. Thus, increasing the success of off-the-shelf conduits will depend upon incorporation of biological cues to stimulate nerve regeneration. Many studies over the last 40 years have investigated the potential of a broad range of molecules and cells to improve peripheral nerve regeneration in animal models with promising results. However, only conduits that are biologically inert have entered the market. Likely reasons for this are (a) the failure of the enhancing agent to prove sufficient benefit in longer nerve gaps, (b) the expense needed for clinical trials and establishing good manufacturing processes (GMP) for the enhancers, and (c) the time consuming and costly regulatory approval pathway.

Tegaserod would be advantageous in these respects. The concentrations needed to promote nerve regeneration are orders of magnitude lower than those administered for irritable bowel syndrome in humans: 2–12 mg/day or 0.025–0.15 mg/kg for an 80 kg patient for a 4–6 week application time as opposed to 46 pg in a single on-site dose in a 20 g mouse, comparable to 2 × 10^−6^ mg/kg, for administration to an injured peripheral nerve. Application of tegaserod at lower than clinically used concentrations could avoid the need for replication of toxicological and pharmacological profiles. In the context of nerve regeneration, tegaserod would be delivered in a single on-site dose, either encapsulated within a scaffold as used in this report or preferably slowly released from a biodegradable drug-eluting conduit. On-site delivery via the conduit should also greatly minimize the likelihood of the very rare adverse coronary effects reported for daily oral administration of high doses of tegaserod (Busti et al., 2004; Schiller and Johnson, 2008). As a previously FDA approved drug with established GMP, commercial translation may likewise be facilitated. Thus, tegaserod has the potential to be repurposed for the application not only of peripheral nerve repair, but hopefully also for amelioration of central nervous system disabilities where PSA is likely to play an important therapeutic role.

## 5. Conclusions

Glycans, such as PSA, play an important role in nervous system development, synaptic plasticity and regeneration following injury. The application of small molecule compounds that mimic the beneficial activity of glycans, such as also the HNK-1 carbohydrate, for nervous system repair may prove to be valuable additions to treatments that rely on other classes of compounds. Also, evaluating approved drugs for alternate mechanisms of action may open novel approaches to pharmacological actions.

## Abbreviations

CDR: complementary determining region
cGN: cerebellar granule neuron
DRG: dorsal root ganglion
ELISA: enzyme-linked immunosorbent assay
FBA: foot base angle
GMP: good manufacturing processes
H: heavy chain
HRP: horse radish peroxidase
L: light chain
NCAM: neural cell adhesion molecule
NIH: National Institutes of Health
PBS: phosphate buffered saline, pH 7.3
PLL: poly-l-lysine
PLO: poly-l-ornithine
PLR: protraction limb ratio
PNS: peripheral nervous system
PSA: polysialic acid
RI: recovery index
SPR: surface plasmon resonance
5-HT: serotonin (5-hydroxytryptamine)
